# Unique coexistence of dispersion stability and nanoparticle chemisorption in alkylamine/alkylacid encapsulated silver nanocolloids

**DOI:** 10.1038/s41598-018-24487-9

**Published:** 2018-04-17

**Authors:** Keisuke Aoshima, Yuya Hirakawa, Takanari Togashi, Masato Kurihara, Shunto Arai, Tatsuo Hasegawa

**Affiliations:** 1 Department of Applied Physics, The University of Tokyo, Tokyo, 113-8656 Japan; 2 National Institute of Advanced Industrial Science and Technology (AIST), AIST Tsukuba Central 5, Tsukuba, 305-8565 Japan; 3 Department of Material and Biological Chemistry, Yamagata University, Yamagata, 990-8560 Japan

## Abstract

Surface encapsulation of metal nanoparticles (NPs) is fundamental to achieve sufficient dispersion stability of metal nanocolloids, or metal nanoink. However, the feature is incompatible with surface reactive nature of the metal NPs, although these features are both essential to realizing the functional applications into printed electronics technologies. Here we show that two different kinds of encapsulation for silver NPs (AgNPs) by alkylamine and alkylacid together are the key to achieve unique compatibility between the high dispersion stability as dense nanoclolloids and the AgNP chemisorption printing on activated patterned polymer surfaces. Advanced confocal dynamic light scattering study reveals that an additive trace amount of oleic acid is the critical parameter for controlling the dispersion and coagulative (or surface-reactive) characteristics of the silver nanocolloids. The composition of the disperse media is also important for obtaining highly concentrated but low-viscosity silver nanocolloids that show very stable dispersion. The results demonstrate that the high-resolution AgNP chemisorption printing is possible only by using unique silver nanocolloids composed of an exceptional balance of ligand formulation and dispersant composition.

## Introduction

Metal nanocolloids (NCs) attract considerable attentions recently as “functional inks” to manufacture fine metal wiring patterns by using printing-based device production technologies^1–4^. Printed electronics are expected to innovate the production of flexible, large-area, and ambient electronic devices without using a vacuum or photolithography^2–5^. Metal NCs are composed of ligand-encapsulated metal nanoparticles (NPs) that can be densely and stably suspended in dispersion media through the ligand encapsulation of active or unstable metal NP surfaces^6,7^. A wide variety of metal NCs has been developed, so far, with different combinations of metals and ligands^1–4^. However, a guiding principle has not yet been established to select and optimize the formulation of functional metal NCs for their use in printed electronics technologies^1–11^.

Among them, a class of silver NCs (AgNCs), obtained by thermal decomposition of oxalate-bridging silver alkylamine complexes, is shown to exhibit both highly stable dispersion as AgNCs and particular self-sintering characteristics after drying of the AgNCs^8^. These AgNCs are composed of silver NPs (AgNPs) (with an average diameter of about 13 nm) encapsulated by alkylamines and a slight amount of oleic acid, and can be suspended at a very high concentration of 40 wt% in a 4:1 (volume ratio) mixed dispersant of *n*-octane and *n*-butanol. Viscosity of these AgNCs is as low as that of the mixed dispersant. The peculiar nature of these AgNCs is that the high dispersion stability is preserved for several months at room temperature, whereas the AgNPs are readily self-fused with each other, eventually exhibiting metallic conductivity even at room temperature, if the metal NC ink is dried (i.e., the dispersant evaporated). These characteristics are seemingly incompatible in terms of the degree of ligand encapsulation, and have never been identified in other classes of metal NCs^9^. Note that similar but different “built-in” self-sintering effects were reported for electrosterically stabilized AgNCs that include diluted electrolytes^10,11^: The AgNPs encapsulated by polyacrylic acid are self-sintered on dried concentration of NaCl electrolytes that act as a destabilizer against encapsulation. In striking contrast, a different self-sintering mechanism should be responsible in the former AgNCs, as they do not include a destabilizer or electrolytes.

Recently, a groundbreaking printing technique was reported by taking advantage of the unique nature of the AgNCs, showing the self-sintering characteristics as presented above^12–14^. The technique is based on an AgNP-chemisorption effect on activated patterned polymer sufaces, which allows easy, high-speed, and large-area manufacturing of ultrafine metal wiring, and thus begins to produce great impact on industrial applications. The technique is only composed of simple two-step processes: the first step is the fabrication of a patterned activated surface by masked vacuum ultraviolet (VUV) irradiation of the perfluoro-polymer layer surface, and the second step is blade coating to expose the AgNCs on the patterned activated surface for a short period of time (less than 1 sec) at room temperature. It is demonstrated by the authors^12^ that the AgNPs included in the AgNCs are exclusively and rapidly adsorbed on the patterned activated surface during the coating process, which enables the production of an invisible ultrafine silver pattern with a minimum line width of 0.8 μm that is conductive without any post-heating treatment, and strongly adheres on the substrate surface. The printing technique is thus quite advantageous in terms of the quality, resolution, and facile nature of the production and is in striking contrast to the conventional printing technique, which has required an additional drying process and post-sintering process for the deposited AgNCs. Nevertheless, a simple question arises regarding the mechanism of the above-mentioned AgNP-chemisorption printing technique, or more intuitively associated with the surface reactivity of suspended AgNPs: Why could the high dispersion stability of the AgNPs be maintained as AgNCs for such a long period of time as long as a few months^12,15,16^, whereas a high reactivity is possible for exclusive and rapid AgNP chemisorption only on the patterned activated surfaces?

In this study, we present the dispersion characteristics of the AgNCs as investigated through the use of advanced dynamic light scattering (DLS) technique^17^ to clarify the origin of the unique compatibility of the dispersion stability and the AgNP-chemisorption printing technique. For this purpose, we utilize a confocal DLS technique^18–20^ to investigate the mobile nature of the AgNPs in dense AgNCs (60 wt% at most) that take the form of a deeply dark black fluid and would be difficult to be probed using the conventional DLS. With this technique, we successfully reveal how the dispersion stability changes with time, and how the dispersion stability and high-resolution printability is affected by the change in the ligand formulation and dispersant composition in the AgNCs. On the basis of the obtained results, we demonstrate that these AgNCs are composed of a unique balance of ligand formulation and dispersant composition, which has been unexplored but should take crucial roles in the high-resolution AgNP-chemisorption printing technique.

## Results

### Two kinds of encapsulation by alkylamine and alkylacid

**Figure 1:**
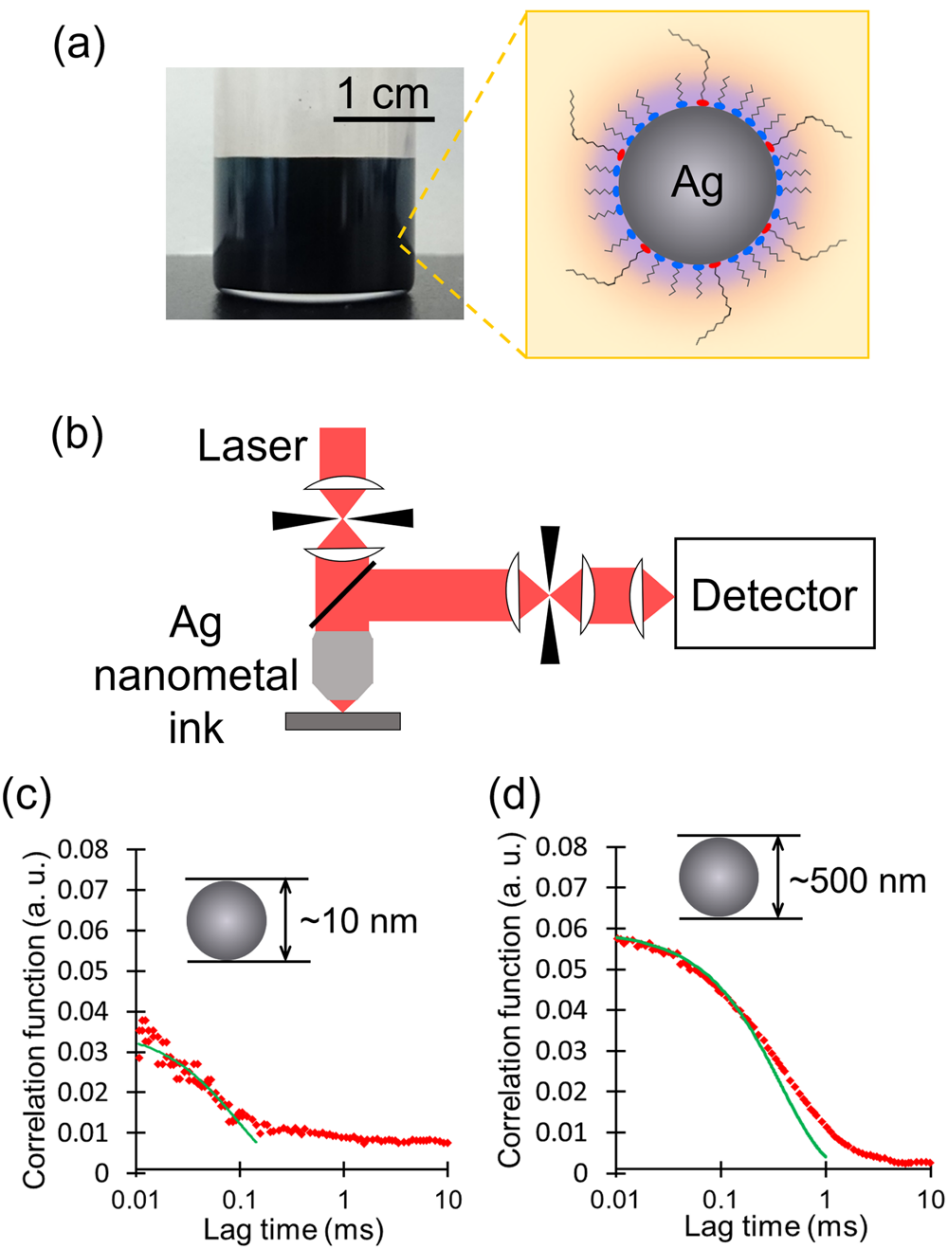
Confocal DLS study on dense AgNCs. (**a**) Synthesized 40 wt% AgNC (left) and a schematic of an encapsulated single AgNP (right). (**b**) Schematic of the confocal DLS system. (**c**) Example time autocorrelation function measured 30 min after the synthesis of the 40 wt% AgNC^*opt*^. (**d**) Example time autocorrelation function measured 3 months after the synthesis of the 40 wt% AgNC^*opt*^. The guide lines in (**c**) and (**d**) are the results of fitting with an exponential decay function. The fast decay between 0.01–0.1 ms in (**c**) and the slow decay between 0.1–1 ms in (**d**) demonstrate the Brownian motions of orginal AgNPs (with particle size of about 10 nm) and flocculated secondary particles (with particle size of about 500 nm), respectively.

Two types of ligand molecules are used for encapsulating the AgNPs in the AgNCs, as schematically presented in Fig. 1a; the major one is short alkylamines that bond relatively weakly with silver via the lone pair of nitrogen atoms, and the minor one is a trace amount of oleic acids that bonds more strongly with silver as oleate anions^21^. These ligand molecules are incorporated into the AgNPs during the synthetic process using thermal decomposition of oxalate-bridging silver complexes^8^. In the synthesis, all the ligand molecules are first added to the oxalate-bridging silver complexes and then heated at 110 °C. The thermal decomposition of the complexes produces AgNPs with a uniform diameter of about 13 nm, and part of the added ligand molecules are chemically bonded to the surface of AgNPs. Excess ligand molecules are then removed from the product by centrifugation, and the final product of encapsulated AgNPs is dispersed in a 4:1 (volume ratio) mixed dispersant of *n*-octane and *n*-butanol to obtain the AgNCs at a concentration of 40 wt%.

The additive amount of oleic acid during the synthesis is much smaller than that of alkylamines; the molar ratio with oxalate-bridging silver complexes is as small as 0.035–0.052 for oleic acid, and as large as 3.8–3.9 for alkylamines. Nonetheless, the oleic acid is found to work quite effectively as a functional encapsulating ligand in the AgNCs, as it takes crucial roles in the high dispersion stability of the AgNCs as well as in the high-resolution printability by the AgNP-chemisorption printing technique, as discussed later. So we finely adjusted the additive amound of oleic acid to obtain the “optimized AgNC” (which we refer as AgNC^*opt*^) that shows highest dispersion stability (See Methods).

We use thermogravimetry and differential thermal analysis (TG/DTA) measurements, in order to identify the amount of oleic acid attached effectively to the AgNPs. The results are presented in Supplementary Fig. 1a and b. We find that the oleic acid molecules detached at a temperature higher than 211 °C, as evidenced by the mass loss in the TG/DTA curves. The total mass loss above 211 °C is linearly correlated with the initial additive amount of oleic acid, being about one tenth of the additive amount, as presented in Supplementary Fig. 1c. Eventually, the number of encapsulating oleic acid molecules is estimated to be about 136 per single AgNP in the AgNC^*opt*^, whose number ratio is 1/46 to the surface silver atoms of the single AgNP. It is natural to assume that the rest surface silver atoms should be encapsualted by alkylamines, as schematically presented in Fig. 1a. This consideration is also demonstrated by the surface enhanced Raman spectrum from the AgNCs as presented in Supplementary Fig. 2.

### Long-term stability of optimized AgNCs

**Figure 2:**
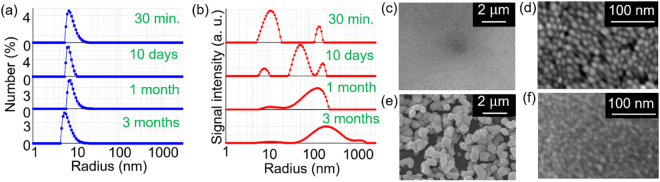
Long-term dispersion stability and coagulation of optimized AgNCs. (**a**,**b**) Size distributions of the AgNC^*opt*^ measured by confocal DLS. The ordinates of (**a**) and (**b**) are the particle number fraction and intensity of the scattered light obtained by an analysis of the measured time autocorrelation function, respectively. The result of (**b**) clearly indicates that the aggregation of AgNPs progress with time. (**c**–**f**) SEM images of the dried surface of the AgNC^*opt*^; (**c**) and (**d**) are those dried after 1 day has passed as a colloid after the synthesis, while (**e**) and (**f**) are those after 3 months have passed as a colloid after the synthesis.

We investigate the dispersion stability of the AgNC^*opt*^ using the confocal DLS system^18–20^ shown schematically in Fig. 1b. Examples of the observed time autocorrelation function of the scattered light from the AgNC^*opt*^ are presented in Fig. 1c and d, measured 30 min and 3 months after the synthesis, respectively. A clear variation is observed in the autocorrelation time by the measurements after different time lapses. The analyzed particle size distributions in the AgNC^*opt*^ measured after different time lapses are shown in Fig. 2a and b; the former presents the particle number fraction as a function of the particle size, and the latter indicates the particle size dependence of the scattered light intensity before the correction to obtain the former (whose time autocorrelation function is indicated in Fig. 1c,d, Supplementary Fig. 3a and b). As seen in Fig. 2a, a clear peak is observed around the original particle radius of 7 nm for 3 months postpreparation. This indicates that the number fraction is dominated by the AgNPs that have the independently dispersed original particle size and demonstrates the high dispersion stability of the AgNC^*opt*^. In contrast, there is a marked change in the particle size distribution of scattered light intensity over time as shown in Fig. 2b; the small additional peak around a particle radius of 120 nm is observed 30 min after preparation in addition to the original peak. This small peak corresponds to clusters composed of several thousand AgNPs as secondary particles. As time passes, a larger peak is observed around a radius of 50 nm after 10 days, around 100 nm after 1 month, and around 200 nm after 3 months. The growth of the additional peaks is accompanied by a decrease in the original peak height at around 10 nm. Note that the different nature of the two-mode particle-size analyses between Fig. 2a and b is primarily ascribed to the considerably large particle size dependence of the scattering cross section. From Fig. 2b, we conclude that the AgNPs flocculate gradually in AgNC^*opt*^, and secondary particles become larger as time passes. The mass fraction of the AgNPs that are flocculated to form secondary particles is estimated as 8.6% after 30 min, 43.5% after 10 days, 72.9% after 1 month, and 97.3% after 3 months.

Figure 2c–f present scanning electron microscope (SEM) images of the dried surface (after a 30 min drying process) of the AgNC^*opt*^ one day and 3 months after synthesis. The AgNC^*opt*^ dried one day after the synthesis exhibits a uniform surface (Fig. 2c), whereas the AgNC^*opt*^ dried 3 months after the synthesis has a granular surface with a particle size of 500–1000 nm (Fig. 2e). These results are well consistent with the particle size distribution as observed in Fig. 2b. The expanded SEM image for the AgNC^*opt*^ dried 3 months after the synthesis as presented in Fig. 2f also indicates that the original AgNPs are retained in the original particle shape (see Fig. 2d) within the flocculated secondary particles even 3 months after synthesis.

### Effects of ligand formulation on the AgNCs

**Figure 3:**
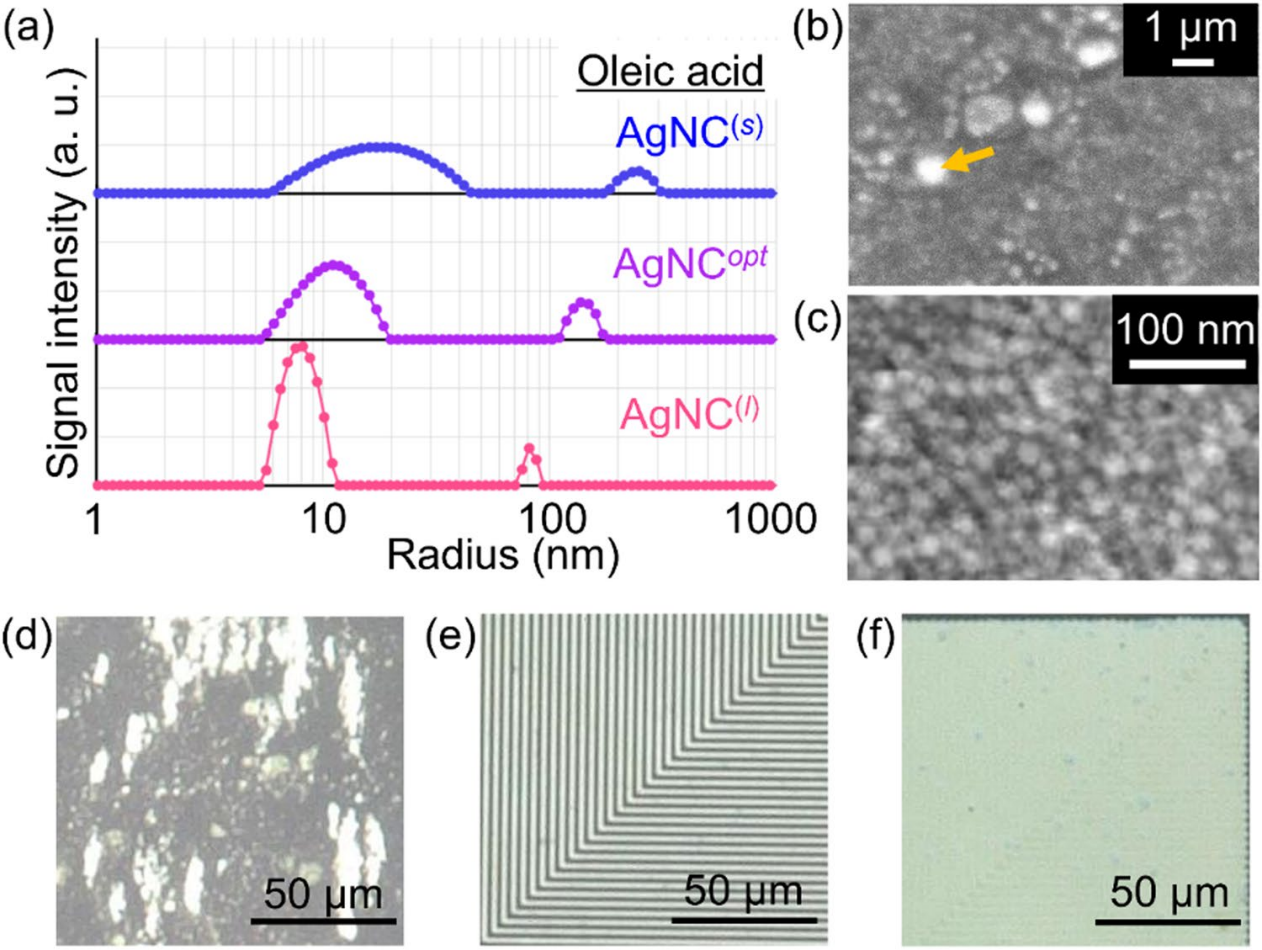
Dependence of AgNC characteristics on the ligand formulation. (**a**) Change in the particle size distribution depending on the additive amount of oleic acid in the Ag nanometal ink: AgNC with smaller amount of oleic acid (AgNC^(*s*)^), AgNC with optimized amount of oleic acid (AgNC^*opt*^), and AgNC with larger amount of oleicacid (AgNC^(*l*)^). (**b**,**c**) SEM images of the dried surface of the AgNC^(*s*)^. In (**b**), the uneven surface with bumps reflects the flocculation of AgNPs. (**c**) Enlarged view of the area indicated by the arrow in (**b**). Numerous single AgNPs that have not perfectly sintered are seen in (**c**). (**d**–**f**) Silver patterns printed with the AgNP-chemisorption printing technique. Patterns formed with (**d**) AgNC^(*s*)^, (**e**) AgNC^*opt*^, and (**f**) AgNC^(*l*)^. Each ink is coated on the same photoactivated surface pattern with a 800 nm line width and space. Only AgNC^*opt*^ forms the ultrafine silver pattern.

To investigate the effect of the ligand formulation on the dispersion stability, we prepare three kinds of AgNCs that are synthesized by different additive amounts of oleic acid: (1) AgNC^*opt*^, (2) AgNC^(*s*)^ with 77% of the optimal amount of oleic acid for fabricating the AgNC^*opt*^ to obtain AgNCs with a smaller amount of oleic-acid encapsulation, and (3) AgNC^(*l*)^ with 115% of the optimal amount of oleic acid to obtain a larger amount of oleic-acid encapsulation. Figure 3a shows the particle size distribution of the scattered light intensity for these AgNCs measured 30 min after synthesis. It is found that as the amount of oleic acid decreases, the peak distribution at the original particle size of about 10 nm broadens considerably, and the secondary peak of the flocculated AgNPs shifts to a larger particle size greater than 100 nm. The results imply that the addition of oleic acid is crucial to suppress AgNPs flocculation in the AgNCs. Figure 3b and c present SEM images of the dried surface of the AgNC^(*s*)^; the latter is expanded view of the former around the particle surface indicated by the arrow. The dried surface as observed in the image is partially composed of some clusters with a size of several hundred nanometers, which is slightly larger than the secondary particle size of 200–300 nm as observed by the DLS measurements shown in Fig. 3a. It is most probable that the AgNPs should be considerably aggregated during the drying process of AgNC^(*s*)^.

**Figure 4:**
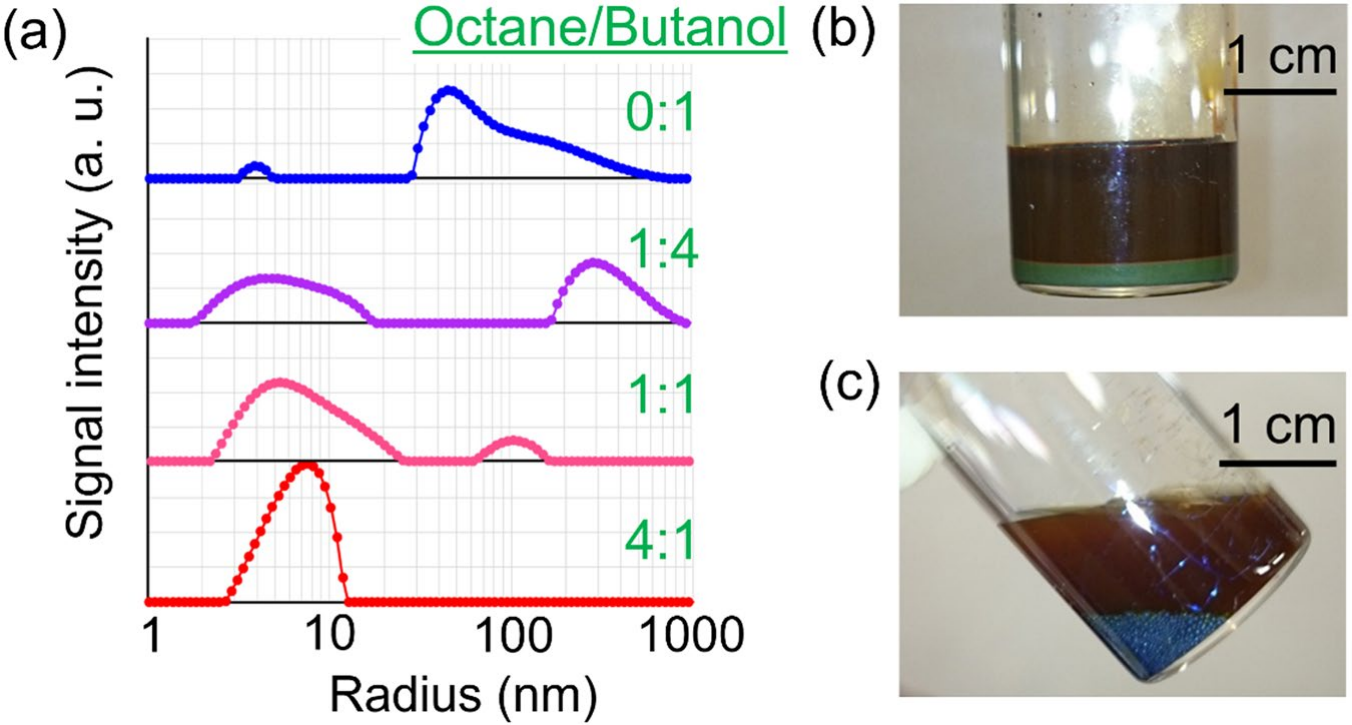
Dependence of AgNC characteristics on the dispersant composition. (**a**) Change in the particle size distribution depending on the composition of the dispersant solvent in AgNCs. Solvents are pure butanol and octane/butanol mixed solvents of volume ratios of 1:4, 1:1, and 4:1 (optimal composition). Clearly from the particle size distribution, the optimal composition solvent is the best dispersion medium. (**b**) Appearance of the AgNCs prepared with pure butanol. AgNCs coagulated and precipitated. (**c**) Appearance of the AgNCs prepared with pure octane. AgNCs coagulate and the phase is separated.

**Figure 5:**
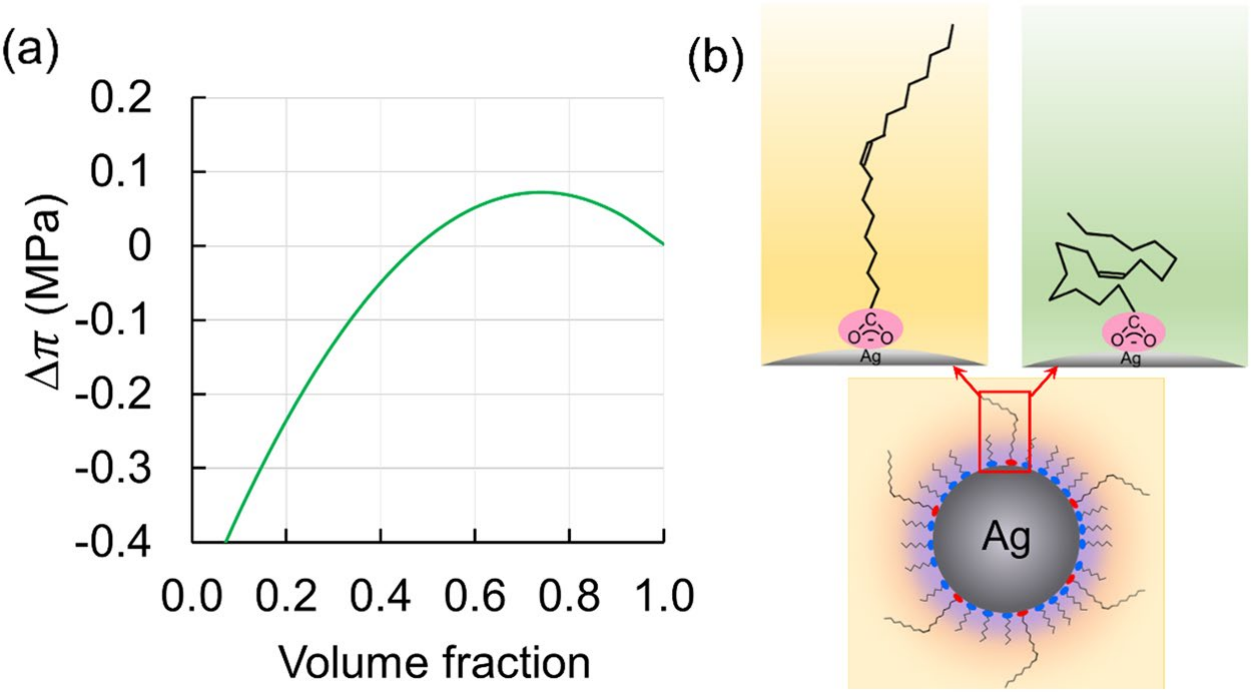
Possible mechanism for the unique dispersion and coagulative characteristics of AgNCs. (**a**) Change in the osmotic pressure between two AgNPs depending on the octane/butanol mixing ratio of the solvent. The abscissa is the volume fraction of octane in the mixed solvent. The ordinate is the calculated increase in the osmotic pressure induced by the approach of AgNPs. (**b**) Proposed image of the ligand oleic acid molecules attached to the AgNP surface in (left) the optimal dispersant and (right) a poor dispersant.

We also investigate the applicability of these AgNCs for use in the AgNP-chemisorption printing technique. For this purpose, we prepare photoactivated surfaces with simple stripe patterns of various linewidths. We find that it is possible to form lines with all the AgNCs (i.e., AgNC^*opt*^, AgNC^(*s*)^, and AgNC^*opt*^), if the line width is larger than 10 μm. However, the fineness of the printed pattern as well as the electrical conductivity is greatly affected by the change in the ligand formulation, as presented in Fig. 3d,e and f. Ultrafine silver patterning with a minimum line width of 800 nm is only possible with the AgNC^*opt*^, as shown in Fig. 3e. In the case of the AgNC^(*s*)^ shown in Fig. 3d, aggregates of the AgNPs with a size of 1–5 μm are sparsely adhered on the substrate surface, which considerably deteriorates the ultrafine silver pattern. Although pattern formation is possible with a line width larger than 10 µm, these aggregates are observed to give nonuniform line patterns with larger line widths, as presented in Supplemenatry Fig. 4. In contrast, in the case of the AgNC^(*l*)^, line patterns with a rather high uniformity are obtained, but the pattern boundary becomes more obscured, or a larger line width is often obtained, as seen in Supplemenatry Fig. 5a and b, which demonstrates a clear contrast with the case of AgNC^*opt*^ as presented in Supplemenatry Fig. 5c. The linewidth tends to become a few micrometers wider, as compared to that of the original photoactivated surface pattern. Thus, the ultrafine pattern with a line width of 800 nm is squashed, as presented in Fig. 3f.

Additionally, a considerably large difference is observed in the electrical conductivity of the printed silver patterns for different ligand formaution of the AgNCs. The average conductivities of the silver electrodes (without post-annealing process) are estimated to be 10^−2^–10^−1^ S/m for the AgNC^(*s*)^, 10^3^–10^5^ S/m for the AgNC^*opt*^, and 10^−1^–10^1^ S/m for the AgNC^(*l*)^. It is found that only printed silver lines fabricated with the AgNC^*opt*^ exhibit a sufficiently high electrical conductivity. It is most probable that the poorer conductivity in the case of AgNC^(*s*)^ should come mainly from the derioration in layer uniformity within the printed silver lines, but not from the slight difference of ligand encapsulation, as discussed later.

### Effects of dispersant composition on the AgNCs

To investigate the effect of the dispersant composition on the dispersion stability, we measure the particle size distribution of the scattered light intensity for four types of AgNCs prepared with different dispersant compositions: (1) pure butanol, and mixed solvents of octane and butanol with mixing ratios of (2) 1:4, (3) 1:1, and (4) 4:1. (The composition (4) is the optimized composition for AgNC^*opt*^.) The additive amounts of alkylamine and oleic acid are constant for all the AgNCs, with values equal to those for producing the AgNC^*opt*^. Figure 4a presents the particle size dependence of the scattered light intensity for the four types of AgNCs. The particle size distribution in the (4) AgNC^*opt*^ consists of a single peak at around 7 nm. In contrast, considerable broadening of that peak and additional peaks appear in size distributions of the other AgNCs. In fact, the AgNC (1) composed of pure butanol is very unstable as a colloid, and presents a precipitation soon after (within 15 min) the synthesis, as presented in Fig. 4b. Similarly, when pure octane is used as the disperse medium, AgNC could not be formed, but two phases are present, as shown in Fig. 4c. These results indicate that the flocculation tendency of the AgNPs is considerably enhanced in the AgNCs with different dispersant compositions other than AgNC^*opt*^. The results are also consistent with the SEM observations of the dried surfaces of these AgNCs (Supplemenatry Fig. 6). We conclude that the dispersion stability of the AgNCs is considerably affected by the mixing ratio of the dispersant even with use of AgNPs synthesized with the same ligand formulation.

## Discussion

The results presented all the above clearly demonstrate that both the ligand formulation of AgNPs and the dispersant composition of AgNCs play critical roles in suppressing flocculation of AgNPs and thus in retaining the dispersion stability of AgNC^*opt*^ that otherwise shows unique self-sintering characteristics between AgNPs after drying at room temperature. It is natural to consider that these two parameters should be closely correlated with each other for suppressing the AgNP flocculation. The following scenario can be considered in regard to the unique dispersion stability of the AgNC^*opt*^: Ligand coordination of the oleic acid on the AgNPs should give rise to suitable repulsive forces between the AgNPs in the 4:1 mixed dispersant of octane and butanol, which effectively prevents the flocculation. The repulsive force could be basically described by the osmotic pressure for the oleic acid ligands in the mixed solvents^15,16^, depending on the similarity (or affinity) between the ligand and the solvent molecules in terms of size, polarity, and hydrogen bonding^22,23^. The accessible distance between the mobile AgNPs within the AgNCs should be directly associated with the oleic acid concentration, so that a change in the dispersion stability of the AgNCs should be caused by a change in the osmotic pressure for the oleic acid ligands in the mixed solvent.

The osmotic pressure, Δ*π*, should depend on the octane/butanol mixing ratio in the mixed solvent. On the basis of classical Flory–Huggins theory^24,25^, we estimated the dependence of Δ*π* on the mixing ratio of the mixed solvents; the result is shown in Fig. 5a. It can be seen that Δ*π* shows a maximum when the volume fraction of octane is equal to about 0.74. This reasonably accounts for the results shown in Fig. 4a, where the high dispersion stability of the AgNCs is only obtained with the 4:1 mixed dispersant. It can also be seen in Fig. 5a that Δ*π* presents a negative value when the volume fractions of octane are smaller than about 0.48. This means that the affinity between the oleic acid and butanol is so low that the flocculation of AgNPs is much accelerated by the phase separation of the oleic acid and the solvent. As an intuitive picture, we suggest that the repulsive forces between the AgNPs are sensitive to the rigidity of the oleic acid ligands, where the stretching state (Fig. 5b left) and folding state (right) of the ligand molecules should depend on the disperse media and take critical roles in the presence or absence of the steric repulsion between the AgNPs.

We also would like to discuss the origin of the unique relationship between the AgNC composition and the ultrafine silver pattern printed by the AgNP-chemisorption printing technique, as presented in Fig. 3d–f and Supplementary Fig. 5. It is demonstrated in a previous report that conductive, ultrafine silver patterns can be manufactured through the exclusive chemisorption of AgNPs on the patterned activated surfaces^12–14^. All the results presented in this report clearly demonstrate that the unique high-resolution AgNP-chemisorption printing mechanism works effectively only with the AgNC^*opt*^ but not with AgNCs with different ligand formulation or different dispersant composition. In the AgNC^*opt*^, the average number of oleic acid molecules that encapsulate a single AgNP is roughly estimated to be 136. The oleic acids may afford effective repulsive forces between the AgNPs in the AgNCs, but not when it is dried during the course of silver line formation. Furthermore, the alkylamines afford dominant encapsulation for the AgNP surface, but are weak and are gradually eliminated by the fusion reaction between AgNPs during the course of sliver line formation. An excess amount of oleic acid in the AgNC^(*l*)^ should result in weaker AgNP chemisorption on the activated surface as well as poorer conductivity of the printed silver line pattern because of the relatively enhanced encapsulation. It is probable that the deposition of the AgNC^(*l*)^ on the patterned activated surface might be rather close to the case of wet/dewet patterning. On the other hand, a scarce amount of oleic acid in the AgNC^(*s*)^ should result in the instability of the dispersion as AgNCs. We consider that the aggregates of AgNPs (or the flocculated AgNPs) with a size of 200–300 nm should be readily generated in the AgNC^(*s*)^, as presented in Fig. 3a, which might also exhibit relatively weak chemisorption on the patterned activated surface. These aggregates should further form larger clusters with sizes of 1–5 µm during solvent evaporation after the printing deposition, as observed in Fig. 3d and Supplemenatry Fig. 4. In this case, the electrical conductivity should deteriorate considerably because of the sparse packing of the large silver clusters on the patterned activated surfaces, as was found in the conductivity measurements. It means that the difference in conductivity should come mainly from the difference in layer uniformity within the printed silver lines.

To summarize, we have investigated the requirements for the composition of extremely superior functional AgNCs that allow high-resolution AgNP-chemisorption printing technique. Based on the confocal DLS measurements, it is found that the combination use of two different types of ligands, i.e., alkylamines and oleic acid, is fundamental to realize the high dispersion stability of AgNC^*opt*^. Especially, a tiny optimized amount of oleic acid for encapsulating AgNPs plays critical roles in achieving the coexistence of dispersion stability and AgNP chemisorption. Furthermore, the use of mixed dispersant of *n*-octane and *n*-butanol is indispensable to obtain highly concentrated and stable AgNCs. We conclude that the solubility of coordinated oleic acid in the mixed dispersant plays essential roles in the repulsive nature between AgNPs that allows stable colloidal dispersion as well as in the self-sintering characteristics of the dried AgNCs. The concept of the combination encapsulation as revealed in this study should be applicable to a wide range of metal nanocolloids, and we believe that the finding of unique compatibility between colloidal dispersion and surface reactivity will constitute a major step towards the vast field of nanoscience and nanotechnology, in addition to the application into printed electronics technologies.

## Methods

### Synthesis of AgNCs

AgNPs have been synthesized by thermal decomposition of oxalate-bridging silver alkylamine complexes, as reported in the literature^8^. The silver oxalate Ag_2_(C_2_O_4_) is first activated by alkylamines (R-NH_2_) via the formation of the oxalate-bridged silver complex [(R-NH_2_)Ag(*μ*-C_2_O_4_)Ag(R-NH_2_)]. The complex is then heated at 110 °C to undergo low-temperature decomposition with evolution of CO_2_, and the alkylamine-encapsulated AgNPs are obtained in an almost quantitative yield. As the alkylamines, we use a combination of *N*,*N*-dimethyldiaminopropane, butylamine, hexylamine, and dodecylamine, with an addition of a trace amount of oleic acid. For preparing the AgNCs composed of different compositions, additive amounts of oleic acid that are finely or slightly different were prepared. The additive amount of oleic acid in each ink is finely adjusted to 20, 26 (optimal composition), or 30 mg per 1000 mg of the final product of AgNCs, to obtain AgNC^(*s*)^, AgNC^*opt*^, and AgNC^(*l*)^, respectively. The AgNPs are spherical in shape, with a mean diameter of 13.6 nm (radius of 6.8 nm). Finally, AgNCs are produced by dispersing the dried AgNPs in a 4:1 mixed solvent of *n*-octane and *n*-butanol at a concentration of 40 wt%. In addition, we prepare four other types of Ag nanometal ink with different dispersant composistions; pure butanol and three octane/butanol-mixed solvents, whose volume ratios are 1:4, 1:1, in addition to the optimal composition of 4:1. In these Ag nanometal inks with different dispersant compositions, the additive amount of protection group molecules is same with the optimal composition (i.e. AgNC^*opt*^). Although we also attempt to prepare Ag nanometal ink having pure octane as a solvent, the AgNPs are not dispersed at all in the solvent.

### Estimation of the amount of oleic acid in the AgNCs

We use TG/DTA measurements to identify the amount of oleic acid that encapsulate the AgNPs. A sample of approximately 10 mg of AgNC is dried for about 2 h under ambient conditions to obtain 4 mg of dried AgNPs. The sample is placed on an aluminum pan set in the furnace of the TG/DTA apparatus (SSC/5200, Seiko Instruments Inc.). The sample pan and a reference pan are heated at a heating rate of 10 °C/min while nitrogen gas flows through the chamber at 200 cc/min. Examples of the obtained TG/DTA curves for the AgNC^(*l*)^ and AgNC that do not include oleic acid are shown in Supplemenatry Fig. 1. A comparison of the curves indicates that the weight loss observed at temperatures higher than 211 °C should be ascribed to the detachment of oleic acid molecules from the AgNP surfaces. Additionally, the total mass loss above 211 °C is linearly correlated with the additive amount of oleic acid, being about one tenth of the additive amount of oleic acid.

### Viscosity of the solvent

We accurately measure the viscosity of the solvent by an electromagnetically spinning (EMS) viscometer^26–28^, as it is necessary to calculate the size distribution of particles based on the analyses of confocal DLS measurements. An aluminum circular plate floating on the sample solvent revolves under a rotating external magnetic field (90 rpm), and the rotational speed of the plate gives the sample viscosity. Low viscosities can be measured by using a small floating plate, since the viscous torque applied to the plate overcomes the frictional force at a sufficiently small radius.

### Confocal DLS measurement

To evaluate the particle size distribution in the dense AgNCs, we construct the confocal DLS apparatus as shown in Fig. 1b, using a variety of optical components including lens, pinholes, mirror, half mirror, variable neutral density (VND) filter, beam diffuser (BD), and sample holder (all manufactured by Sigma Koki Inc., Japan). As the light source, we use a semiconductor laser (*λ* = 638 nm; LuxX 638–100, Omicron). The laser light is focused on to the sample AgNC through an objective lens (ELWD Plan 50x/0.55, Sigma Koki Inc.). The sample AgNC is placed into a rectangular glass vial with inner dimensions of 1 mm × 10 mm × 50 mm, which is fixed in the optical system by a sample holder. To prevent heating of the samples, the laser power is set to a value below 1 mW. The time variation in the backscattered light from the sample is detected by an avalanche photodiode and a multiple-tau digital real-time correlator unit (ALV-7004/USB-FAST, ALV) for 90 sec; the autocorrelation function of the backscattered light is then calculated. The autocorrelation function of the detected scattered light intensity *I*(*t*) can be expressed as follows^15,16,24^;

1 $$\frac{\langle I(t)I(t+\tau )\rangle }{{\langle I(t)\rangle }^{2}}=1+\exp (-2D{q}^{2}\tau ),$$

where *τ* is the delay time, *q* is a scattering vector, and *D* is the diffusion coefficient. The particle radius *a* can be obtained from *D* using the following Stokes–Einstein equation^15,16,24^,

2 $$D=\frac{{k}_{B}T}{6\pi \eta a},$$

where *k*_B_ is the Boltzmann constant, *T* is the absolute temperature, and *η* is the viscosity of the solvent. In this analysis, the autocorrelation function is analyzed with the CONTIN method^29^, and the size distribution of the particles in the sample AgNC is obtained. Note that this size distribution is a histogram of the scattered light intensity. Therefore, when discussing the number distribution or mass fraction, the distribution is corrected, in consideration of the particle size dependence of the scattered light intensity. Under these measurement conditions, Rayleigh scattering should occur from the particles with size of 200 nm or less. In this case, the scattered light intensity is proportional to the sixth power of the particle radius (*I* ∝ *a*^6^). In contrast, Mie scattering should occur from the particles with size of 200 nm or more, where the scattered light intensity shows very complicated behavior.

To calibrate the confocal DLS system, we use highly monodispersed colloid solution containing polystyrene beads (<15%) with an average particle radius of 50 nm (Polysciences, Inc.). The observed single peak is roughly consistent with the average radius of the spheres (Supplemenatry Fig. 7).

### SEM evaluation of particle size in dried ink

As reference of the particle size distribution taken by confocal DLS, SEM images of the surface of the dried AgNCs are measured. The drying of the ink is performed under ambient conditions for 30 min. We obtain images with an SEM system equipped with a field-emission gun (JSM-7000 F or –7500 F, JEOL Ltd.) and an acceleration voltage of 25 kV.

### Manufacture of printed silver electrodes

In the AgNP-chemisorption printing technique^12^, we first prepare a patterned photoactivated surface on a substrate. A thin film of amorphous perfluorinated polymer, poly [perfluoro (4-vinyloxy-1-butene)] (Cytop)^30^, is fabricated on a substrate by spin coating a diluted solution in a fluorinated solvent (CTL-809M, Asahi Glass Co., Ltd.) at 2,000 rpm for 60 sec at room temperature. The film is then dried at 180 °C at 0.02 MPa for 60 min. A patterned photoactivated polymer surface is produced by masked VUV irradiation with wavelength of 172 nm using a Xe_2_ eximer lamp (VUS-3150, ORC Manufacturing Co., Ltd.) through a photomask. The film is set in a chamber filled with N_2_ gas with a residual O_2_ lower than 300 ppm. The typical thickness of the Cytop layer is 700 nm. The film is irradiated by VUV light at an average dose of 6.4 mW/cm^2^ for 80 sec.

The patterned photoactivated surface is coated with the AgNCs using a blade-coating technique at a sweep rate of 2 mm/sec under ambient conditions. Eventually, a thin silver layer is deposited only on the photoactivated area. To obtain sufficiently high conductivity, we dry the electrodes for a minimum of 5 h under ambient conditions.

### Observation of the morphology of electrodes

The surface morphology of the electrodes obtained by the AgNP-chemisorption printing technique is characterized using an atomic force microscope (AFM; Dimension^TM^3100, diVMG). We capture the tapping-mode AFM images at a scan rate of 1 Hz.

### Conductivity measurement

For confirmation of the conductivity, simple silver line patterns with a length of 3 mm and widths of 1, 3, 5, 10, 50, and 100 μm (which become 2–5 μm wider in the case of AgNC^(*l*)^) are fabricated with the AgNP-chemisorption printing technique and evaluated.

### Calculation of osmotic pressure between AgNPs

On the basis of the classical Flory–Huggins theory^24,25^, the increase in osmotic pressure Δ*π* between two approaching AgNPs is calculated as;

3 $${\rm{\Delta }}\pi =\frac{2{k}_{{\rm{B}}}T}{{v}_{{\rm{m}}}}(\frac{1}{2}-\chi (T)){\varphi }^{2},$$

where *v*_m_ is the average volume of the solvent molecule, *ϕ* is the volume fraction of oleic acid in the area covered by the oleic acid around the AgNP, and *χ*(*T*) is the Flory–Huggins parameter that expresses the affinity between the oleic acid and the solvent molecule. The affinity between different molecules is determined by the similarity of the molecules in terms of size, polarity, and presence of hydrogen bonding. It is known that the similarity is strongly correlated with the compatibility of the molecules, which is often evaluated using a solubility parameter, *δ*^22,23^, and according to Hansen’s theory, *χ*(*T*) between molecule 1 and 2 is represented in terms of *δ* as^31^;

4 $$\chi (T)=\frac{{v}_{{\rm{m}}}}{4{k}_{{\rm{B}}}T}\{4{({\delta }_{{\rm{D}}1}-{\delta }_{{\rm{D}}2})}^{2}+{({\delta }_{{\rm{P}}1}-{\delta }_{{\rm{P}}2})}^{2}+{({\delta }_{{\rm{H}}1}-{\delta }_{{\rm{H}}2})}^{2}\},$$

where *δ*_D_, *δ*_P_ and *δ*_H_ are the Hansen solubility parameters of each molecule expressing the dispersion, polar, and hydrogen-bonding contributions, respectively. We estimate the Hansen solubility parameters of oleic acid and each solvent in Fig. 5a given in the literature^23^, except for octane which we set to have zero polarity.

### Data availability

The datasets generated during and/or analysed during the current study are available from the corresponding author on reasonable request.

## Electronic supplementary material

Supplementary Figures
