# Supplementary material for: Unique coexistence of dispersion stability and nanoparticle chemisorption in alkylamine/alkylacid encapsulated silver nanocolloids
Source: Sci Rep. 2018 Apr 17;8:6133. doi: 10.1038/s41598-018-24487-9 (PMC5904114; doi:10.1038/s41598-018-24487-9)
Supplement: Supplementary file 1 — Supplementary Figures [file 41598_2018_24487_MOESM1_ESM.doc]

SUPPLEMENTARY INFORMATION

Unique coexistence of dispersion stability and nanoparticle chemisorption in alkylamine/alkylacid encapsulated silver nanocolloids

Keisuke Aoshima,^1,2^ Yuya Hirakawa,^1,2^ Takanari Togashi,^3^ Masato Kurihara,^3^ Shunto Arai,^1,2^ and Tatsuo Hasegawa^1,2*^

**
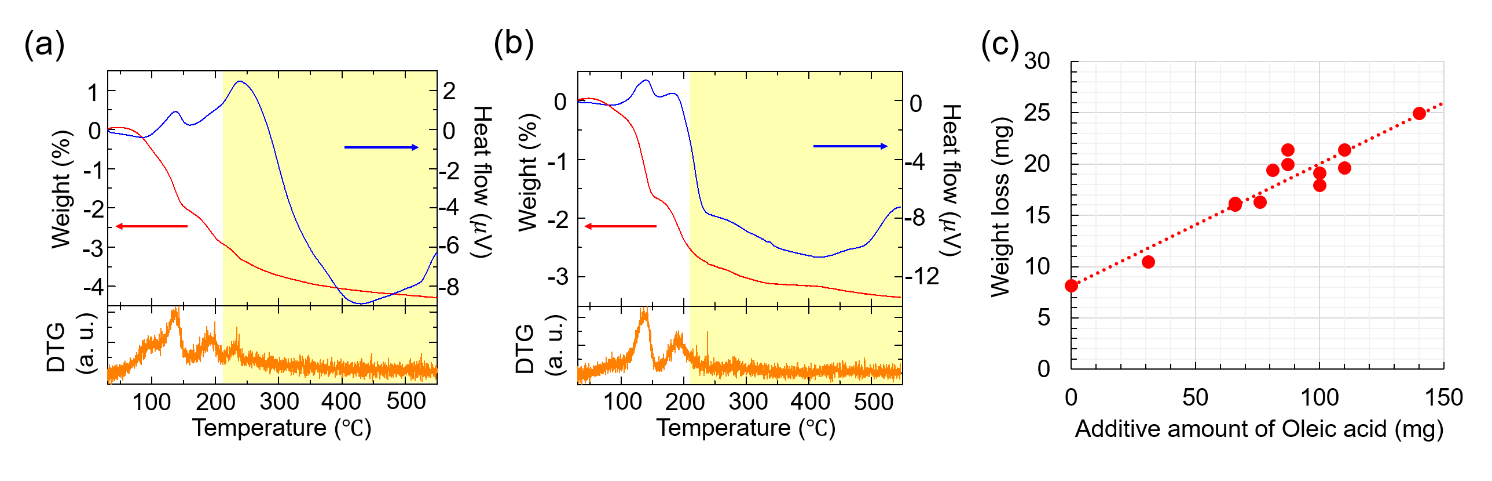
**

**Supplemenary Figure 1 | Thermal analyses of alkylamine/alkylacid-encapsulated AgNPs.** (**a,b**) Example TG/DTA curves of the (**a**) AgNP(*^l^*) and (**b**) AgNP that do not include oleic acid. The upper panels indicate the weight loss (left axis) and heat flow (right axis), and the lower panels indicate the differential weight loss (DTG). A comparison of (**a**) and (**b**) indicates that the weight loss observed at temperatures higher than 211 °C should be ascribed to oleic acid molecules detaching from the AgNPs (yellow region in the graph), while that at temperatures lower than 211 °C should be ascribed to alkylamine molecules. It is probable that the broad exothermic feature observed in (**a**) and (**b**) should be attributed to the self-fusion reaction between AgNPs. (**c**) Amount of oleic acid needed for encapsulating the AgNPs in the AgNCs as estimated by the TG/DTA measurement plotted against the additive amount of oleic acid to produce the AgNCs. Each weight value is that included in 3375 mg of AgNCs. The fit is obtained by a least-squares method. The value of the intercept of the fit is about 8 mg, which should be ascribed to the weight loss of alkylamines detached at temperatures higher than 211 °C.


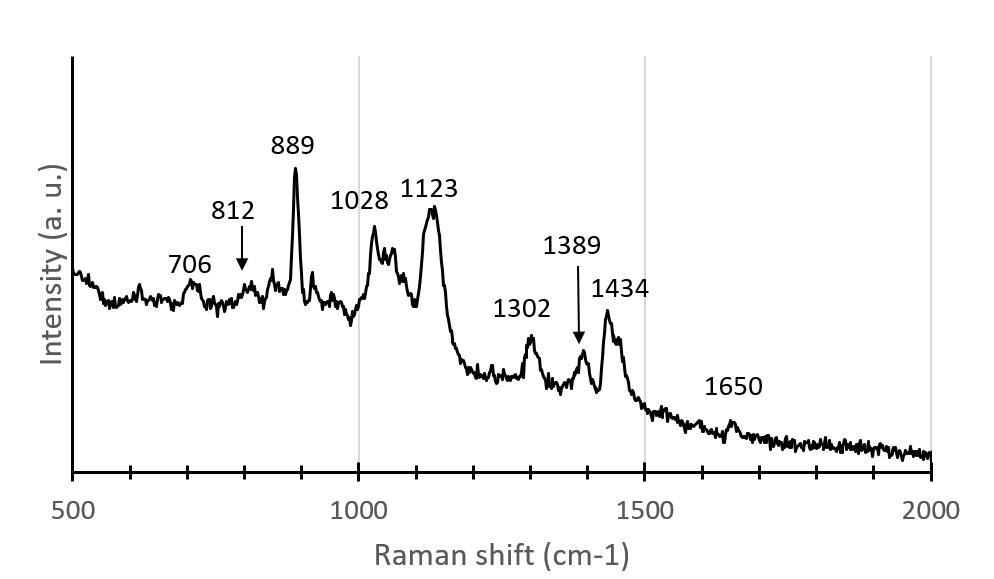


**Supplemenary Figure 2 | Raman spectrum of the AgNC*^op^*.** Surface enhanced Raman spectrum of the 40wt% AgNC*^opt^*. The peak observed at 1,389 cm^−1^ (or 1,434 cm^−1^) can be attributed as COO^−^ stretching vibration of the encapsulating oleic acid, whereas the peak observed at 1,123 cm^−1^ should be associsated with NH_2_ deformation vibration of the encapsulating alkylamines.


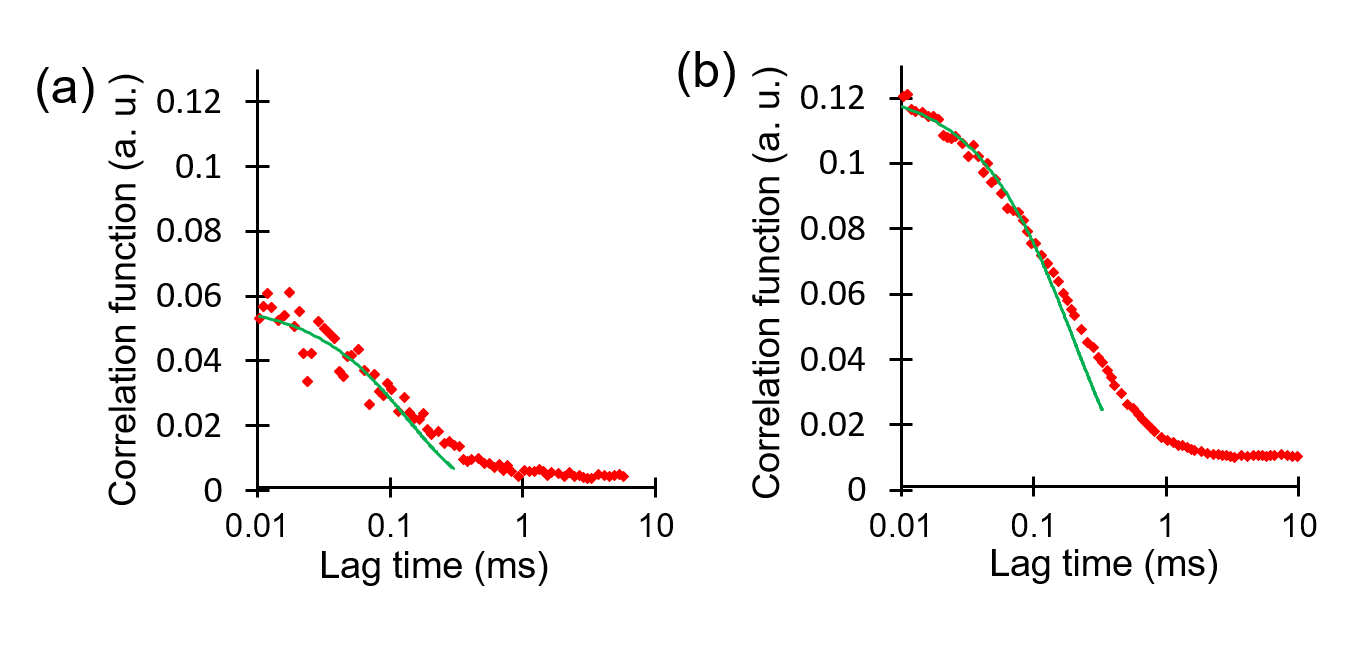


**Supplemenary Figure 3 | Time autocorrelation functions of the AgNC*^opt^*.** Measured time autocorrelation functions (red dots) of the 40wt% AgNC*^opt^* measured (**a**) 10 days and (**b**) 1 month after synthesis. Fits using a single exponential decay function are shown by the green curves.

**
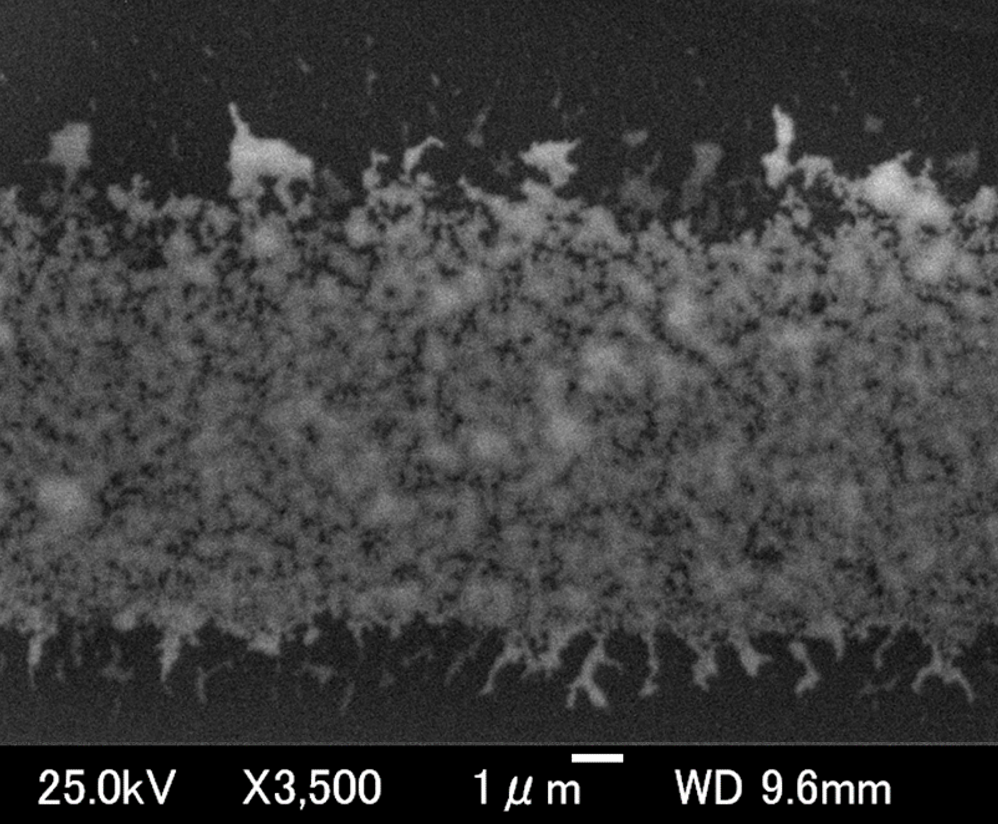
**

**Supplemenary Figure 4 | Silver pattern using the AgNC(*^s^*).** SEM image of the printed silver pattern obtained by the AgNP-chemisorption printing technique using the AgNC(*^s^*) and a photoactivated surface pattern with a line width of 10 μm. The obtained line pattern includes a large inhomogeneity due to clusters of AgNPs with a size of about 1 μm that adhere sparsely on the surface.

**
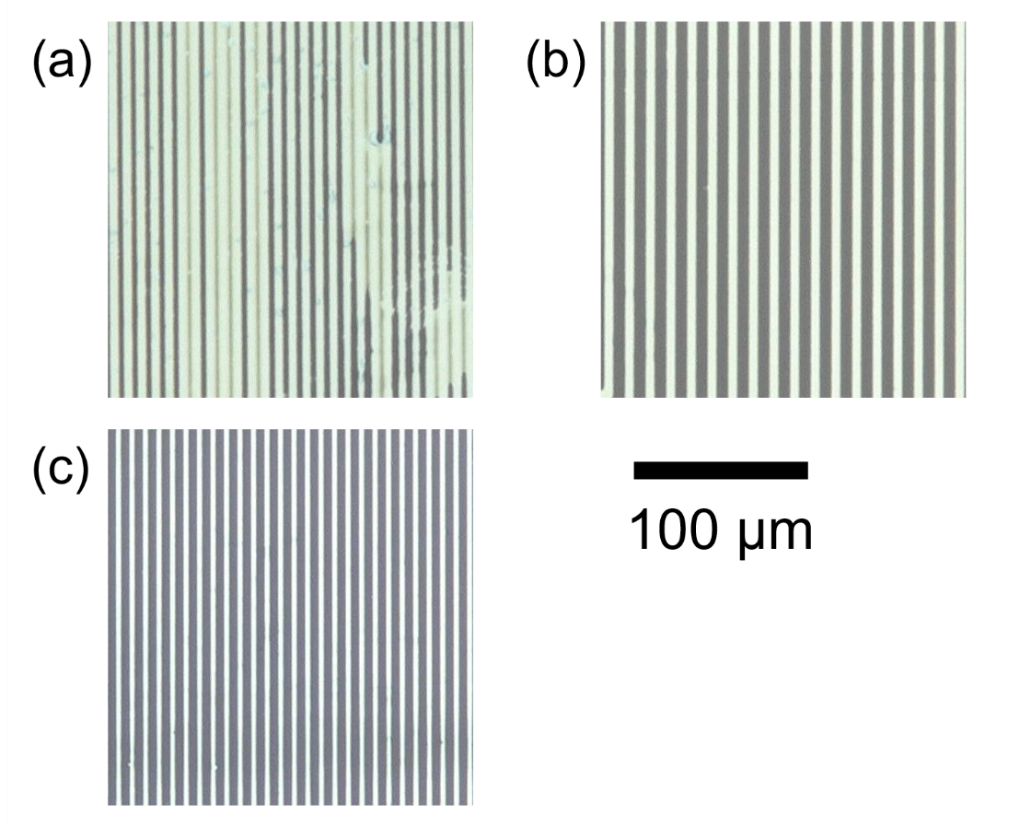
**

**Supplemenary Figure 5 |** **Silver patterns using AgNC(*^l^*) and AgNC*^opt^*.** Optical microscope images of the printed line-and-space silver patterns obtained with the AgNP-chemisorption printing technique using the (**a**) AgNC(*^l^*) and a photoactivated surface pattern with a 6 μm line width and 2 μm space width, (**b**) AgNC(*^l^*) and a photoactivated surface pattern with a 9 μm line width and 3 μm space width, and (**c**) AgNC*^opt^* and a photoactivated surface pattern with a 6 μm line width and 2 μm space width. A comparison of (**a**) and (**b**) with (**c**) demonstrates that high-resolution silver patterning is possible only with the AgNC*^opt^* but not with the AgNC(*^l^*), which tends to afford line patterns whose line width is a few micrometers wider than that of the original photoactivated surface pattern.

**
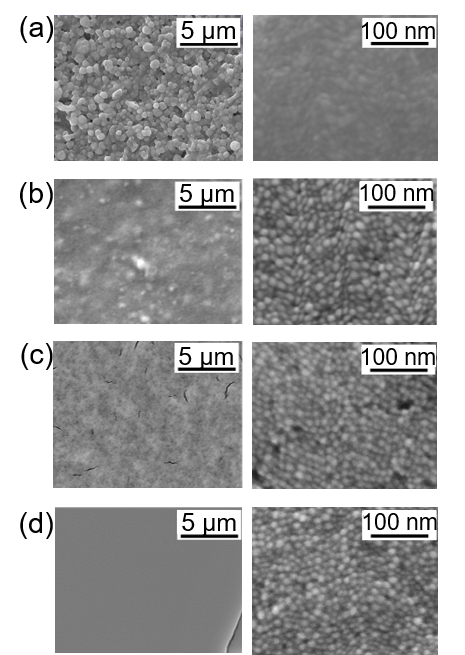
**

**Supplemenary Figure 6 |** **SEM images of the dried AgNCs prepared with different dispersant compositions**. (**a**) pure butanol, and mixed solvents of octane and butanol with mixing ratios of (**b**) 1:4, (**c**) 1:1, and (**d**) 4:1 (optimal composition).

**
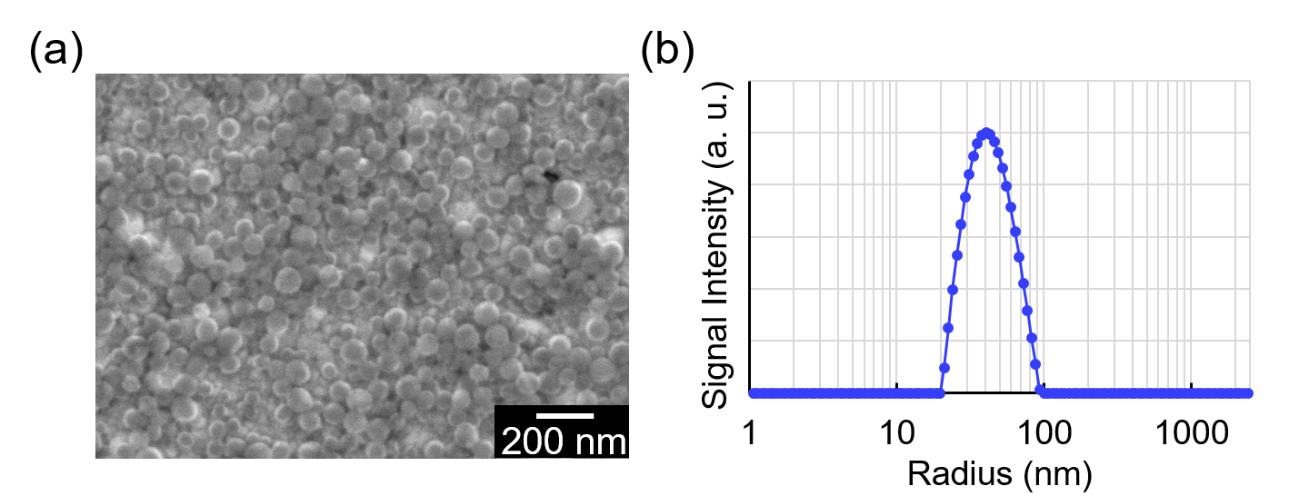
**

**Supplemenary Figure 7 |** **Polystyrene colloid used as the standard.** (**a**) SEM image of the polystyrene beads used for calibrating the confocal DLS system. The average particle radius is estimated to be about 42 nm. (**b**) Result of the confocal DLS measurement of a monodispersed colloid solution containing the polystyrene beads shown in (**a**). The observed single peak is consistent with the particle size.
